# Measuring premenstrual disorders in adolescence: prevalence of premenstrual syndrome and premenstrual dysphoric disorder and psychometric validation of the PMS-I-Y in a German-speaking sample

**DOI:** 10.1186/s12905-026-04752-0

**Published:** 2026-08-15

**Authors:** Jette Geßner, Alexandra Döpke, Luisa Concetta Valenti, Hanna Christiansen, Cornelia Weise

**Affiliations:** 1 Department of Psychology, Clinical Psychology and Behavioral Health Technology, Friedrich-Alexander-Universität Erlangen-Nürnberg, Erlangen, Germany; 2 Department of Psychology, Division of Clinical Psychology and Psychotherapy, Philipps-University Marburg, Marburg, Germany; 3 Deutsches Zentrum für Psychische Gesundheit (DZPG), German Center for Mental Health, Marburg, Germany

**Keywords:** PMS, PMDD, Adolescents, Impact, Assessment, Diagnostics

## Abstract

**Background:**

Premenstrual syndrome (PMS) and premenstrual dysphoric disorder (PMDD) are common conditions associated with significant impairment and distress across multiple domains of functioning, including academic and occupational settings. Although menstruation, and with it the potential onset of PMS or PMDD, typically begins in early adolescence, research in adolescent populations remains limited. Therefore, the present study was conducted to examine the prevalence of PMS and PMDD in German-speaking adolescents, and to evaluate the psychometric properties of the adolescent-adapted version of the Premenstrual Impact Questionnaire (PMS-I-Y).

**Methods:**

In this cross-sectional online study, 521 adolescents aged 14–18 years from Germany, Austria and Switzerland were recruited via Instagram. Premenstrual symptoms were assessed using the PMS-Q-Y. Participants meeting criteria for at least mild PMS completed the PMS-I-Y to assess symptom-related impairment. Severity classifications were based on operationalized questionnaire criteria derived from DSM-5 and PSST-A frameworks. Confirmatory and exploratory factor analyses were conducted to examine factorial validity. Convergent and discriminant validity were assessed using correlations with symptom severity and personality traits.

**Results:**

A substantial proportion of participants reported clinically relevant premenstrual burden, with 46.6% meeting criteria for moderate-to-severe PMS and 22.5% meeting criteria for PMDD. Factor-analytic results indicated a strong general factor of premenstrual impairment alongside psychological and functional components. Convergent validity was supported by strong associations with symptom severity (*r* = .65 − .75). Discriminant validity was indicated by negligible correlations with openness, extraversion, and agreeableness (all *r* ≤ .08), small negative correlations with conscientiousness (*r* = − .11 to − 0.13), and small to moderate positive correlations with neuroticism (*r* = .16 − .33).

**Conclusions:**

Premenstrual symptoms are highly prevalent and associated with substantial psychological and functional burden in adolescents. The PMS-I-Y, an adolescent-adapted version of an existing instrument, shows promising psychometric properties for assessing premenstrual impairment in this age group. However, findings should be interpreted with caution due to the use of retrospective self-report measures, potential sampling bias resulting in a self-selected sample, and the lack of prospective diagnostic assessment. Accordingly, prevalence estimates reflect retrospective symptom reporting operationalized questionnaire criteria rather than clinical diagnoses.

## Introduction

Premenstrual symptoms are commonly experienced across the reproductive lifespan and are associated with a range of psychological and physiological changes, including mood swings, depressive episodes, breast pain, and fatigue [1]. These symptoms typically emerge during the late luteal phase and subside with the onset of menstruation or within the first days of the new cycle [2]. Overall, 80% to 90% of women report experiencing at least one premenstrual symptom [3, 4]. For some, these symptoms are mild and manageable. However, a significant proportion of women face severe psychological and functional impairment affecting school or work performance, mental health, and everyday activities, in which case PMS or PMDD should be considered. PMS, as defined by the American College of Obstetricians and Gynecologists [5] is characterized by the presence of at least one affective and one physical symptom in the premenstrual phase. In contrast, PMDD represents the most severe form of premenstrual distress, with symptoms resembling those of major depression [6]. In the DSM-5 it is classified as an independent diagnosis within the affective disorders section, whereas the ICD-11 assigns it as a separate diagnosis (GA34.41) primarily to gynecological disorders associated with the menstrual cycle. In addition, there is a cross-reference to PMDD in the chapter on depressive disorders [7]. To meet the DSM-5 diagnostic criteria for PMDD, an individual must experience at least five out of eleven possible symptoms, including at least one core affective symptom (mood swings, marked irritability, marked depressed mood or marked anxiety). Additionally, the symptoms must cause clinically relevant distress or impairment and must be confirmed through prospective daily assessment over two consecutive cycles. Crucially, symptoms typically remit or significantly diminish within the first days of menstruation, although some individuals may experience a more gradual resolution over the early part of the cycle [2, 8]. In the past 50 years, research on PMS and PMDD has expanded considerably. However, adolescents were long overlooked, often presumed to be unaffected or only mildly impacted [9, 10]. This view has shifted, recognizing that symptoms can cause significant morbidity in adolescents [11, 12]. Studies from Asia and Africa highlight the need for increased attention to adolescents, reporting prevalence rates of moderate PMS between 30% and 40% and PMDD from 2% to 12% [13, 14]. Despite these findings, research focusing on adolescents in Western cultures remains limited. Although premenstrual disorders are not culture-bound syndromes, cultural factors influence the frequency, intensity and expression of symptoms as well as help-seeking behavior [11]. To our knowledge, only one German study has assessed the prevalence of PMS and PMDD among adolescents and young adults, reporting rates of 18.6% for moderate to severe PMS and 5.3% for PMDD in individuals aged 14–24 years [15]. However, as this study was conducted over two decades ago and relied on outdated diagnostic tools, its current accuracy is thus uncertain. Given these gaps in literature, understanding the impact of PMS and PMDD on adolescents’ daily functioning and mental health becomes particularly important. Symptoms can impair academic performance (e.g., concentration difficulties, school absenteeism) and affect interpersonal relationships and recreational activities, highlighting the need for further research [16–18]. Additionally, both conditions are linked to an increased risk of comorbidities, including heightened suicidality as well as depression and anxiety disorders [19, 20]. These findings underscore that PMS and PMDD cause substantial distress and impairment in adolescents. Although distress and impairment are key diagnostic criteria for PMDD in the DSM-5 and ICD-11 [21], they are often only superficially addressed in existing questionnaires [22–24]. Most instruments primarily assess functional limitations, such as difficulties in relationships, daily activities, or school and work performance. However, given the well-documented link between PMS, PMDD, and reduced life satisfaction, greater emphasis on the emotional and psychological dimensions in assessment tools is warranted [25]. From a theoretical perspective, assessing symptom-related impairment complements symptom-based approaches by capturing the functional and psychological consequences of premenstrual symptoms. This aligns with broader health frameworks that emphasize not only symptom presence but also the extent to which symptoms interfere with daily functioning and well-being. To address this limitation, Kues et al. [26] developed the PMS-Impact Questionnaire (PMS-I) which systematically captures both functional and psychological impairments. This approach offers several advantages, such as identifying individuals with severe symptoms who may require treatment [26]. Moreover, assessing the most affected areas of life enables more targeted and effective interventions and supports the evaluation of treatment efficacy [26]. Although several instruments assess premenstrual symptoms, fewer tools specifically capture the broader psychological and functional impact of these symptoms, particularly in adolescents. Given developmental differences in emotion regulation, social context, and daily demands, assessing symptom-related impairment may provide additional clinically relevant information beyond symptom presence alone. Despite these advantages, no such instrument has yet been adapted or validated for adolescents, underscoring a critical gap and the need for further research. Importantly, the present study distinguishes between premenstrual symptom presence, symptom-related impairment, and formal diagnostic classification. While premenstrual symptoms are assessed using self-report measures and used to estimate prevalence, these estimates do not constitute clinical diagnoses. In contrast, the PMS-I-Y specifically aims to capture the subjective impact of premenstrual symptoms on daily functioning. This distinction is critical, as symptom presence, perceived impairment, and diagnostic classification represent related but conceptually distinct aspects of premenstrual disorders. This study aimed to estimate the prevalence of self-reported PMS and PMDD and to evaluate the psychometric properties of the adolescent-adapted PMS-I-Y in a German-speaking adolescent sample while conceptually distinguishing between symptom presence, impairment-related burden, and diagnostic classification.

## Methods

### Study design

This study employed a cross-sectional online survey design.

### Procedure and participants

Participants were recruited conveniently via Instagram between August and September 2023. Eligible participants were adolescents aged 14 to 18 years, menstruating and residing in Germany, Austria, or Switzerland.

**Fig. 1:**
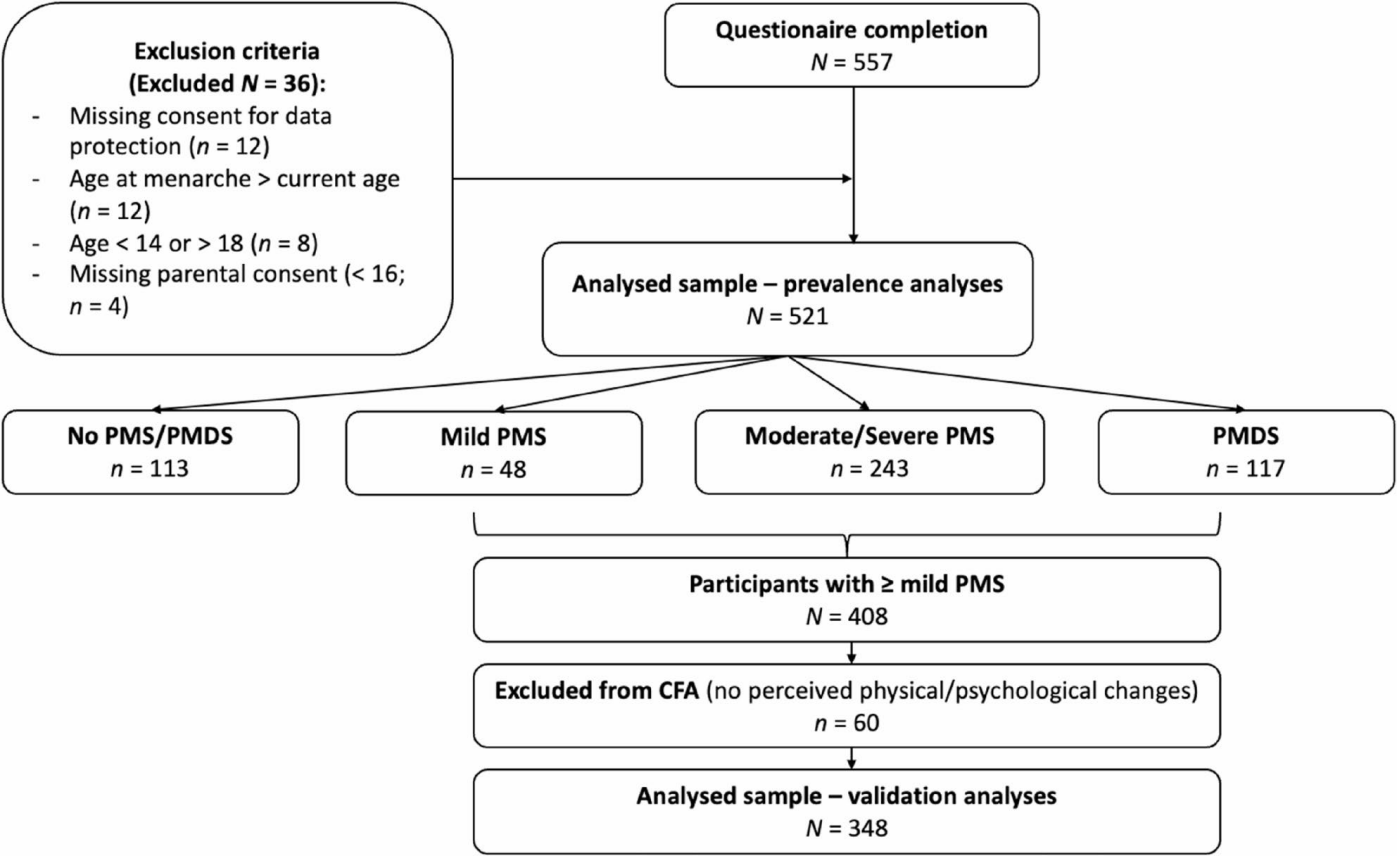
Flow diagram for sample selection for the study

A total of 557 participants completed the initial screening questionnaire. After applying predefined exclusion criteria (e.g. inconsistent age data, missing consent), 521 participants were included in the final sample for prevalence analyses (see Fig. 1).

Participants first completed demographic questions, including age, gender, migratory background, age at menarche, cycle regularity and duration. Premenstrual symptoms were assessed using the adolescent-adapted PMS Questionnaire (PMS-Q-Y) [22]. Participants meeting criteria for at least mild PMS (*n* = 408) completed the Premenstrual Impact Questionnaire for Youth (PMS-I-Y) [26] to assess symptom-related impairment. All participants also completed the Positive Mental Health Scale (PMHS) [27], the Big Five Inventory Short Form (BFI-S) [28], and additional questions on living environment and PMS awareness. They were also invited to provide comments on symptoms, impairment, and desired support. At the end of the study, participants were presented with a recommendation to consult a gynecologist if needed.

### Measures

#### Premenstrual symptoms

Premenstrual symptoms were assessed using the adolescent-adapted version of the DSM-IV-TR based questionnaire on premenstrual syndrome (PMS-Q) [22], referred to as PMS-Q-Y. The PMS-Q-Y is a self-report questionnaire with 30 items, of which 27 assess symptom presence and severity (e.g., “I suddenly start crying”), while three address impairment at school, social activities, and relationships (e.g., “These symptoms affect me in social activities”). Symptoms are rated on a four-point Likert scale (0 = “does not apply” to 3 = “strongly applies”) and impairment from (0 = “not at all” to 3 = “severe”). Although the PMS-Q-Y cannot be used for diagnosing PMS or PMDD, as the DSM-5 requires prospective symptom tracking [8], the instrument effectively estimates prevalence [22]. The adaptation for adolescents involved simplifying language, adding additional instructions, and modifying examples to ensure relevance to school-related demands and adolescent social contexts. Items were reviewed to reflect age-appropriate concerns, particularly regarding school-related impairments and social interactions. To achieve this, the Marburg youth council (https://www.uni-marburg.de/de/fb04/dzpg/mitmachen.

/jugendrat) and other adolescents from the target group were asked to review the scale and provide comments. The original PMS-Q has demonstrated good validity and reliability [22]. In the current study, the PMS-Q-Y showed good internal consistency (*α* = 0.88).

#### Symptom severity classification

Symptom severity levels were classified using criteria derived from both the DSM-5 and the Premenstrual Symptoms Screening Tool revised for Adolescents (PSST-A) [24]. Because the PMS-Q-Y does not provide its own diagnostic cut-offs, severity categories were operationalized based on established DSM-5 and PSST-A criteria, adapted to the PMS-Q-Y item structure. Three severity categories were defined. *Mild PMS* was defined as the presence of at least one symptom rated as “moderate” and at least one impairment rated as “moderate”. *Moderate to severe PMS* required at least one affective symptom and at least four additional symptoms rated “moderate” or “strongly applies” and at least one functional impairment rated “moderately” or “strongly applies”. *PMDD* required ≥ 1 affective symptom and ≥ 4 additional symptoms rated “strongly applies”, and ≥ 1 impairment rated “severe”. This classification approach was chosen to identify adolescents experiencing clinically relevant premenstrual symptom burden, including those who may narrowly miss strict PMDD criteria but still report substantial distress. Supporting this approach, Steiner et al. [23] demonstrated that a considerable proportion of individuals who do not meet full PMDD criteria nonetheless experience marked emotional and functional impairment. Accordingly, the category of moderate to severe PMS was used to capture adolescents with clinically meaningful symptom burden below the PMDD threshold.

##### Premenstrual impact

Impairment associated with premenstrual symptoms was assessed using the adolescent-adapted version of the Premenstrual Impact Questionnaire (PMS-I) [26], referred to as the Premenstrual Impact Questionnaire for Youth (PMS-I-Y). Adjustments were also made with the support of the Marburg youth council (https://www.uni-marburg.de/de/fb04/dzpg/mitmachen/jugendrat*)* and other adolescents from the target group to ensure the tool’s applicability for adolescents. Specifically, language was simplified, gender-neutral wording was implemented, an introduction was added, and the instructions were modified to avoid explicit use of the term “PMS” to reduce potential bias. Examples were modified to better align with adolescents’ lives. In addition, qualitative feedback from adolescents was used to refine item wording and enhance developmental appropriateness. However, no formal pilot testing or cognitive interviewing procedures were conducted prior to the present study, which should be considered when interpreting the findings. The PMS-I-Y consists of 18 items rated on a four-point Likert scale ranging from 1 (“does not apply”) to 4 (“strongly applies”), e.g., “Due to the symptoms before my period, I enjoy leisure activities less”. Higher scores indicate higher distress. The original PMS-I demonstrated good validity [26]. The questionnaire comprises two theoretically derived subscales: Psychological Impact (PI) and Functional Impact (FI).

#### Positive mental health

Positive mental health was assessed using the Positive Mental Health Scale (PMHS) [27] to account for broader psychological well-being. The PMHS is a nine-item self-report questionnaire rated on a four-point Likert scale ranging from 0 (“do not agree”) to 3 (“agree”). Item scores are summed to yield a total score, with higher values indicating higher levels of positive mental health. The PMHS was included to assess general psychological well-being and to help rule out alternative explanations for elevated premenstrual symptom burden. The scale has demonstrated good psychometric properties [27] and showed high internal consistency in the present study (*α* = 0.89).

##### Personality traits

To perform discriminant validation of the PMS-I-Y, the Big Five Inventory Short Form (BFI-S) [28] was used to assess personality traits. Personality traits were chosen as a theoretically distinct, relatively stable trait measure to provide an initial test of discriminant validity. This approach allows us to examine whether premenstrual impairment reflects a state-dependent phenomenon rather than a stable dispositional characteristic. This 15-item self-report inventory measures openness, conscientiousness, extraversion, agreeableness, and neuroticism (three items per trait). The items are rated on a seven-point Likert scale (“does not apply at all” to “fully applies”). The BFI-S has demonstrated acceptable psychometric properties in adults [29], good test-retest reliability over 18 months, and consistent psychometric properties across age groups, supporting its use with adolescents [30]. Internal consistency in the current study was marginally satisfactory (*α* = 0.69).

### Statistical analyses

All analyses were conducted using R Studio (Version 2025.09.1).

### Assessment of prevalence

For prevalence estimation, the percentage of participants meeting criteria for ‘*no PMS’*, ‘*mild PMS’*, ‘*moderate to severe PMS’*, and ‘*PMDD’* based on the PMS-Q-Y was calculated. Severity classifications were derived from an operationalization based on DSM-5 criteria and the PSST-A framework [24], adapted to the PMS-Q-Y item structure, as described above.

### Psychometric analyses

Psychometric analyses of the PMS-I-Y included only participants meeting at least mild PMS criteria who also completed the PMS-I-Y (*N* = 408).

### Reliability

Internal consistency reliability was assessed using Cronbach’s alpha coefficients for the total scale and the two subscales, Psychological Impact (PI) and Functional Impact (FI).

### Construct validity

To evaluate the construct validity of the *Premenstrual Syndrome Impact-Youth (PMS-I-Y) scale*, factor-analytic procedures were conducted. For these analyses, participants were included only if they met criteria for at least mild PMS and did not endorse the item “I do not notice any physical and/or psychological changes in myself” (*n* = 60), resulting in a final factor-analytic sample of *N* = 348. Participants reporting no perceived symptom changes were excluded to ensure sufficient variance in the construct of interest and because they were not expected to meaningfully contribute to the latent structure of premenstrual symptom-related impairment.

### Exploratory factor analysis

Given the mixed findings reported in previous studies and the adaptation of the instrument for adolescents, an exploratory factor analysis (EFA) was first conducted to examine the underlying factor structure of the PMS-I-Y in the present sample. EFAs were based on polychoric correlation matrices and estimated using minimum residual extraction with oblimin rotation, allowing factors to correlate. The number of factors was determined based on eigenvalues, scree plot inspection, factor interpretability, and theoretical considerations.

In addition, exploratory item-reduction sensitivity analyses were performed to investigate whether removing items with low factor loadings or substantial cross-loadings would improve the factor structure and overall model fit.

### Confirmatory factor analysis

Following the EFA, confirmatory factor analyses (CFAs) were conducted to test theoretically derived measurement models. Given the predefined factor structure of the original instrument [26] a confirmatory approach was used to evaluate the fit of the proposed dimensional structure. First, a two-factor model was specified, assigning all PMS-I-Y items to the latent dimensions Psychological Impact and Functional Impact in accordance with the original validation study. Model parameters were estimated using weighted least squares estimation with mean and variance adjustment (WLSMV), which is recommended for ordinal Likert-scale data. Model fit was evaluated using the chi-square test (χ²), Comparative Fit Index (CFI), Tucker-Lewis Index (TLI), Root Mean Square Error of Approximation (RMSEA), and Standardized Root Mean Square Residual (SRMR). Following established recommendations, CFI and TLI values above 0.95 indicate good fit, whereas values above 0.90 indicate acceptable fit. RMSEA values of 0.06 or lower indicate good fit, and values up to 0.08 indicate acceptable fit. SRMR values of 0.08 or lower indicate good fit, while values up to 0.10 are generally considered acceptable [31].

To further examine the dimensional structure of premenstrual symptom-related impairment, a bifactor model was subsequently tested. Following Chen et al. [32], bifactor models are particularly suitable when both a general factor and domain-specific dimensions are of interest. The bifactor model consisted of (a) two orthogonal domain-specific factors representing Psychological Impact and Functional Impact and (b) a general factor representing overall premenstrual symptom-related impairment. The general factor was specified to load directly on all items, while the domain-specific factors accounted for residual variance unique to their respective domains. Covariances between the general and specific factors were fixed to zero, consistent with the orthogonal bifactor framework. Model estimation again used WLSMV, and model fit was evaluated using the same fit indices as the two-factor CFA.

### Convergent and discriminant validity

Convergent and discriminant validity were assessed using Pearson correlation coefficients. Convergent validity was examined by correlating PMS-I-Y total and subscale scores with premenstrual symptom severity measured by the PMS-Q-Y. Moderate to strong positive correlations (*r* ≥ .30) were expected. Discriminant validity was evaluated by examining correlations between PMS-I-Y scores and personality traits measured using the BFI-S. Low correlations (*r* ≤ .10) were expected for most personality traits, whereas small positive correlations were anticipated for neuroticism, based on previous findings [26, 33].

### Descriptive analysis of symptom burden

Finally, the burden associated with premenstrual symptoms was explored descriptively by comparing mean PMS-I-Y item scores to identify domains of impairment perceived as most burdensome by adolescents.

### Ethical considerations

The study was approved by the Ethics Committee at the Department of Psychology at Philipps University Marburg (approval: 2023-53). Written informed consent was obtained from all participants and from parents for those below the age of 16. In accordance with Article 8 of the EU-General Data Protection Regulation (GDPR) and applicable national regulations in Germany, Austria, and Switzerland, adolescents aged 16 years and older are generally considered able to provide independent consent for the processing of personal data in minimal-risk research contexts.

## Results

### Sample characteristics

Sample characteristics of the total sample (*N* = 521) are presented in Table 1.

**Table 1:** Sample characteristics of the study participants (*N* = 521)

Variable	M (SD)	Range / *n* (%)
Age (years)	16.20 (1.34)	14–18
Age at menarche (years)	12.42 (1.20)	10–18
Duration of menstruation (days)	6.13 (3.69)	3–37
Gender		
Female	-	510 (97.89%)
Non-binary	-	7 (1.34%)
Trans-male	-	4 (0.77%)
General mental health (PMHS)	2.90 (0.62)	1–4
BFI-S		
BFI-S Openness	5.20 (1.16)	1–7
BFI-S Conscientiousness	5.03 (1.08)	1.67-7
BFI-S Extraversion	4.30 (1.49)	1–7
BFI-S Agreeableness	5.28 (0.98)	2.33-7
BFI-S Neuroticism	4.90 (1.16)	1–7

*PMHS* Positive Mental Health Scale, *BFI-S* Big Five Inventory-Short Form

### Descriptive statistics of premenstrual symptoms

Descriptive statistics for premenstrual symptom severity and symptom-related impairment are presented in Table 2. Participants reported moderate levels of premenstrual symptoms and impairment overall. Mean scores for the PMS-Q-Y and PMS-I-Y are summarized in Table 2.

**Table 2:** Descriptive statistics for premenstrual symptom severity (*N* = 521) and impairment (*N* = 408)

Variable	M (SD)	Range / *n* (%)
PMS-Q-Y		
PMS-Q-Y Symptoms	2.45 (0.63)	1–4
PMS-Q-Y Impairment	2.69 (0.76)	1–4
PMS-I-Y		
PMS-I-Y Total	2.41 (0.68)	1-3.89
PMS-I-Y Functional Impact	2.25 (0.70)	1-3.89
PMS-I-Y Psychological Impact	2.57 (0.79)	1–4

*PMS-Q-Y * Premenstrual Symptoms Questionnaire-Youth, *PMS-I-Y* Premenstrual Syndrome Impact–Youth

### Prevalence estimates for PMS and PMDD

Based on the PMS-Q-Y and the classification criteria applied in the present study, prevalence estimates for PMS and PMDD were calculated. Of the 521 study participants, 9.2% (*N* = 48) met the criteria for mild PMS, 46.6% (*N* = 243) met the criteria for moderate to severe PMS, and 22.5% (*N* = 117) met the criteria for PMDD. 21.5% (*N* = 113) did not meet the criteria for PMS or PMDD. With regard to premenstrual symptoms assessed using the PMS-Q-Y, participants most frequently reported irritability, sudden feelings of sadness, and changes in eating behavior. These symptoms were also most commonly rated as having a negative impact on school or work performance.

### Psychometric properties of the PMS-I-Y

For the factor analyses, *N* = 348 participants met the inclusion criteria. This subsample had a mean (SD) age of 16.3 (1.3) years, a mean (SD) age at menarche of 12.3 (1.26) years and mean (SD) level of positive mental health was 2.9 (0.62).

### Reliability

Internal consistency of the PMS-I-Y was good to excellent. Cronbach’s alpha was 0.90 for the total scale, 0.85 for the Functional Impact subscale, and 0.87 for the Psychological Impact subscale, indicating good internal consistency of the instrument.

### Construct validity

Construct validity was evaluated using exploratory and confirmatory factor analyses.

### Exploratory factor analysis

Exploratory factor analyses were conducted to examine the underlying structure of the PMS-I-Y in the adolescent sample. A one-factor solution (Table 3) indicated a strong general factor, with all items loading positively (factor loadings ranging from 0.31 to 0.76) and explaining 40% of the variance; however, model fit was insufficient (RMSEA = 0.146, TLI = 0.657). A two-factor solution (Table 4) roughly differentiated between psychological and functional impairment, explaining 50% of the variance. The factors were moderately correlated (*r* = .52), and several items showed cross-loadings, indicating limited discriminant validity. Model fit improved but remained suboptimal (RMSEA = 0.098, TLI = 0.846). A three-factor solution explained 53% of the variance, but the third factor accounted for only 4% of additional variance and showed weak and unstable loadings (e.g., 0.33-0.35) and low factor determinacy (minimum correlation = 0.01). Model fit showed only marginal improvement (RMSEA = 0.093, TLI = 0.861). Overall, the EFA results suggest a strong general factor with limited evidence for clearly separable subdimensions. These findings are consistent with the bifactor model described above, which demonstrated superior overall model fit.

**Table 3:** Exploratory factor analysis (one-factor solution) of the PMS-I-Y (*N* = 348)

Item	Factor Loading (MR1)	h^2^	u^2^
PI02_07	0.76	0.57	0.43
PI02_18	0.72	0.52	0.48
PI02_13	0.70	0.49	0.51
PI02_15	0.68	0.47	0.53
PI02_01	0.68	0.47	0.53
PI02_16	0.67	0.45	0.55
PI02_03	0.67	0.45	0.55
PI02_04	0.67	0.45	0.55
PI02_05	0.66	0.44	0.56
PI02_09	0.64	0.41	0.59
PI02_08	0.63	0.40	0.60
PI02_12	0.63	0.39	0.61
PI02_10	0.63	0.39	0.61
PI02_17	0.62	0.38	0.62
PI02_02	0.60	0.36	0.64
PI02_14	0.51	0.26	0.74
PI02_11	0.46	0.21	0.79
PI02_06	0.31	0.10	0.90

Factor loadings are based on a one-factor solution using minimum residual (minres) extraction. h^2^ = communality; u^2^ = uniqueness. Items are sorted by factor loading

**Table 4:** Exploratory factor analysis (two-factor solution) of the PMS-I-Y (*N* = 348)

Item	MR1	MR2	h^2^	u^2^
PI02_18	0.91	-	0.76	0.24
PI02_10	0.80	-	0.58	0.42
PI02_08	0.77	-	0.55	0.45
PI02_07	0.74	-	0.65	0.35
PI02_16	0.69	-	0.53	0.47
PI02_15	0.61	-	0.50	0.50
PI02_13	0.59	-	0.51	0.49
PI02_02	0.52	-	0.38	0.62
PI02_11	-	-	0.21	0.79
PI02_05	-	0.90	0.73	0.27
PI02_03	-	0.84	0.68	0.32
PI02_17	-	0.67	0.50	0.50
PI02_14	-	0.64	0.39	0.61
PI02_04	-	0.63	0.52	0.48
PI02_01	-	0.52	0.49	0.51
PI02_09	-	0.51	0.44	0.56
PI02_06	-	0.44	0.17	0.83
PI02_12	-	0.38	0.39	0.61

Factor loadings < 0.30 are suppressed. h² = communality; u² = uniqueness. Items are grouped by their primary loading. PI02_12 shows cross-loadings on both factors. PI02_11 did not show a salient loading on either factor

### Exploratory item-reduction sensitivity analyses

Exploratory item-reduction sensitivity analyses were conducted to examine whether removing weakly loading or cross-loading items improved model fit. Compared with the original 18-item two-factor model (CFI = 0.985, TLI = 0.982, RMSEA = 0.067, SRMR = 0.070), reduced models showed only modest improvements. The most reduced 14-item model, excluding PI02_06, PI02_11, PI02_12, and PI02_01, showed the best fit (CFI = 0.991, TLI = 0.990, RMSEA = 0.058, SRMR = 0.062), while internal consistency remained high (α = 0.891). However, because item removal was exploratory and may affect content validity, the original 18-item version was retained for the main analyses.

### Confirmatory factor analysis

**Fig. 2:**
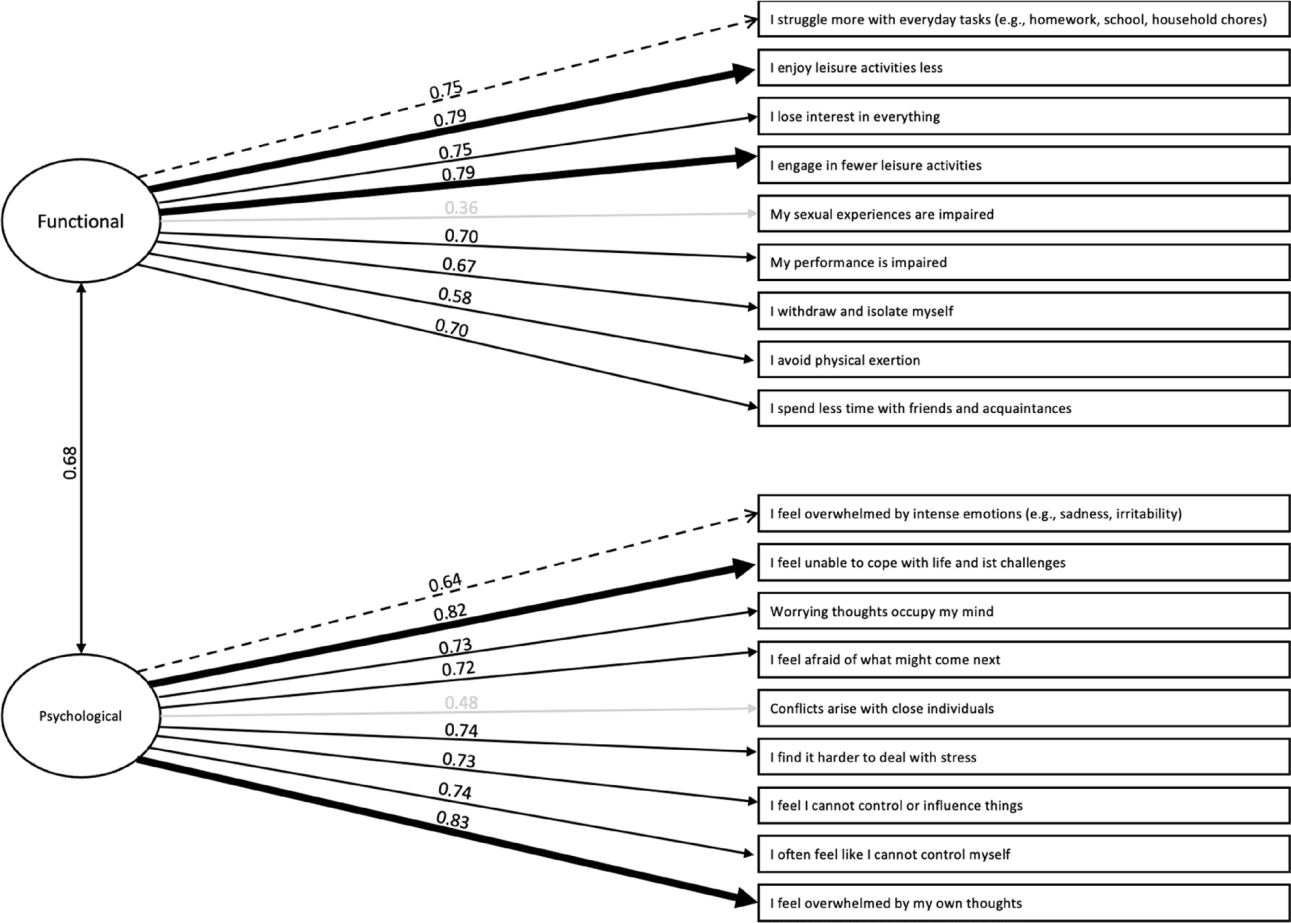
Standardized factor loadings of the two-factor confirmatory factor analysis model of the PMS-I-Y, comprising psychological impact and functional impact

In a second step, a two-factor model was estimated based on the factors Psychological Impact and Functional Impact. The chi-square test indicated a significant deviation from perfect model fit (*χ*² (134) = 343.05, *p* < .001). However, given the sensitivity of *χ*² statistic to sample size, additional fit indices were examined, suggesting an overall acceptable fit according to most fit indices (CFI = 0.99, TLI = 0.98, SRMR = 0.07), although the RMSEA value (0.07) indicated only limited fit. All items loaded significantly on their respective latent factors, with standardized loadings ranging from 0.36 (item PI02_06) to 0.83 (item PI02_18). The correlation between Psychological Impact and Functional Impact was strong (*r* = .68, *p* < .001). Standardized factor loadings of the two-factor model are presented in Fig. 2.

Rectangles represent observed variables; circles represent latent factors. Solid arrows indicate factor loadings; double-headed arrows indicate correlations between latent factors.

### Bifactor analysis

**Fig. 3:**
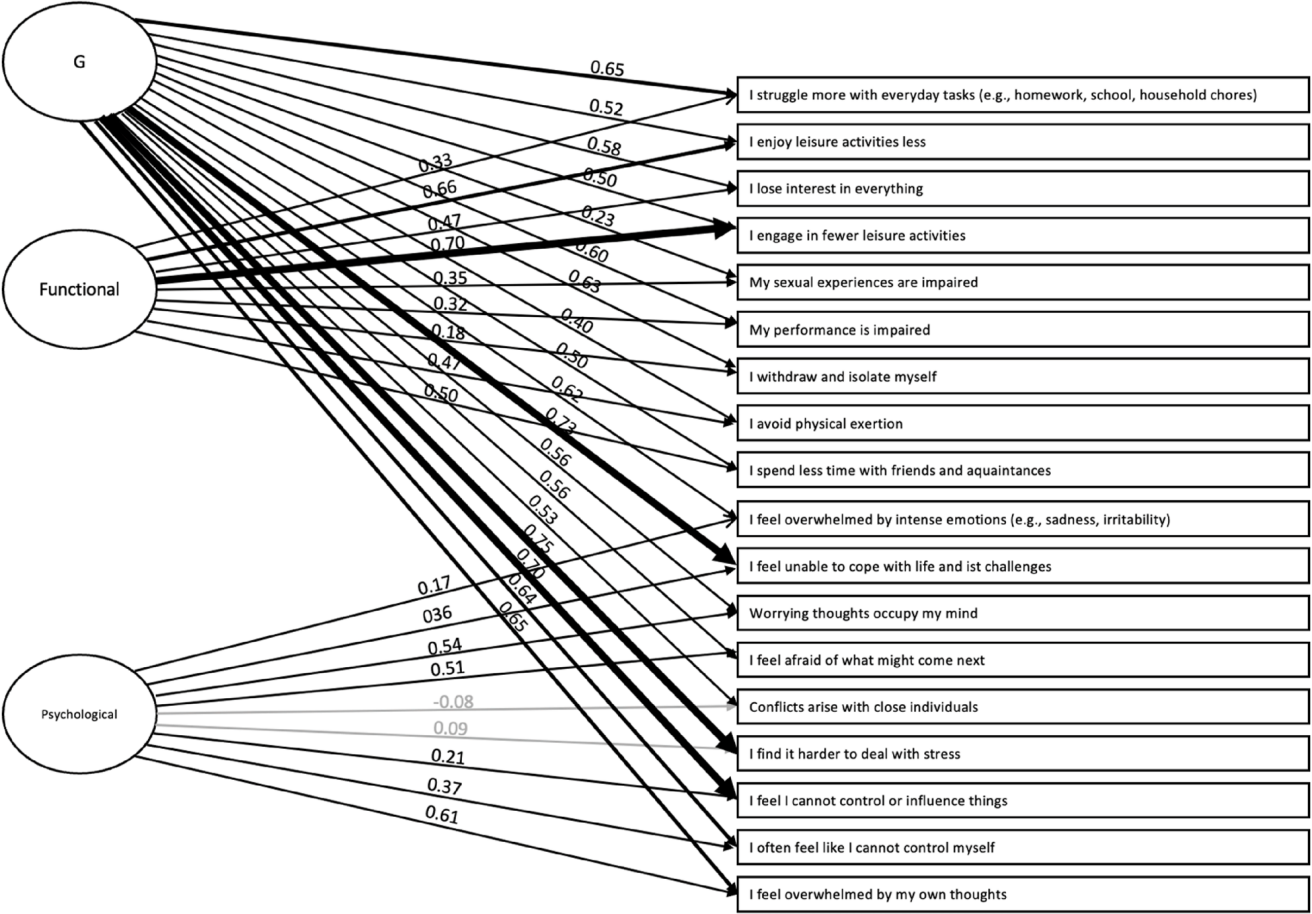
Standardized factor loadings of the bifactor model of the PMS-I-Y, including a general impact factor and the domain-specific factors psychological impact and functional impact

Given the mixed evidence for the two-factor model and to further explore the dimensional structure of the premenstrual symptom-related impairment, a bifactor model was estimated. This model specified a general impact factor (G) alongside the two domain-specific factors Psychological Impact and Functional Impact. The bifactor model showed an overall good to excellent fit. Although the chi-square test was significant (χ²(117) = 165.47, *p* = .002), additional fit indices indicated a very good model fit (CFI = 0.996, TLI = 0.995, SRMR = 0.048, RMSEA = 0.035, 90% CI [0.021, 0.046]). All items loaded significantly on the general factor, with standardized loadings ranging from 0.23 (item PI02_06) to 0.75 (item PI02_13). The domain-specific factors Psychological Impact and Functional Impact also showed several significant loadings; however, some psychological impact items (e.g., PI02_11, PI02_13) exhibited weak or non-significant loadings, suggesting that a substantial proportion of variance in these items was captured by the general factor. As specified, covariances between the general and specific factors were fixed to zero, reflecting the orthogonal bifactor structure. Standardized factor loadings for the bifactor model are presented in Fig. 3.

Rectangles represent observed variables; circles represent latent factors. Solid arrows indicate factor loadings; double-headed arrows indicate correlations between latent factors.

### Convergent validity of the PMS-I-Y with the PMS-Q-Y

Convergent validity was supported by strong correlations between premenstrual symptom severity assessed with the PMS-Q-Y and premenstrual symptom burden and impact assessed with the PMS-I-Y. High correlations were observed for the PMS-I-Y total score (*r* = .75, *p* < .001) as well as for the Psychological Impact subscale (*r* = .68, *p* < .001) and the Functional Impact subscale (*r* = .65, *p* < .001).

### Discriminant validity of the PMS-I-Y with the BFI-S

Discriminant validity was examined by correlating PMS-I-Y scores with personality traits assessed using the BFI-S. No significant correlations were found between PMS-I-Y total or subscale scores and the personality traits openness to experience, extraversion, and agreeableness (all *rs* ≤ 0.08, all *ps* ≥ 0.18). Small negative correlations were observed with conscientiousness (*r*_total = − 0.13, *r*_FI = − 0.11, *r*_PI = − 0.12; all *ps* ≤ 0.04). In contrast, small to moderate positive correlations were found with neuroticism (*r*_total = 0.28, *r*_FI = 0.16, *r*_PI = 0.33; all *ps* < 0.001).

## Discussion

This study aimed to address the limited research on PMS and PMDD in adolescents in German-speaking countries and to evaluate the suitability of the PMS-I-Y for assessing the burden of these disorders in this age group.

### Prevalence of symptoms

The prevalence of moderate to severe PMS observed in this study (46.6%) aligns with international reports from adolescent samples, which have documented prevalence rates ranging from 30% to 50% [13, 34, 35]. However, these estimates are substantially higher than those reported in earlier studies from Western populations, which have typically ranged between 18.6% and 31% [15, 24]. Importantly, the prevalence rates reported in the present study should be interpreted with caution. Given the retrospective self-report design, the use of operationalized questionnaire-based criteria, and the absence of prospective symptom tracking, these findings are unlikely to reflect true diagnostic prevalence. Instead, they more plausibly indicate elevated levels of self-reported premenstrual symptom burden within the sample. Earlier community-based studies [15] relied on diagnostic frameworks and assessment instruments that were not specifically adapted to adolescents and focused primarily on strict DSM-based PMDD criteria, which may have contributed to more conservative prevalence estimates in younger populations. In contrast, although Steiner et al. [22] employed an adolescent-adapted screening instrument (PSST-A), their approach primarily targeted severe PMS and PMDD and may have been less sensitive to moderate or subthreshold but clinically relevant symptom burden. The present study used a transparent and theoretically sound classification based on the DSM-5 and PSST-A criteria, adapted to the PMS-Q-Y, potentially capturing a broader spectrum of clinically relevant premenstrual symptom burden. The comparatively small proportion of adolescents without premenstrual symptoms (21.5%) is noteworthy, as it represents the smallest subgroup in the sample. This distribution suggests that premenstrual symptoms are common, rather than the exception, even in adolescence, highlighting the need for further research in this age group. The PMDD prevalence of 22.5% observed in this study is substantially higher than the previously reported rates of 2.2% to 12% in adolescent samples [13, 14]. This finding should be interpreted with caution, as PMDD diagnoses according to DSM-5 require prospective symptom tracking and exclusion of alternative mental disorders, while ICD-11 likewise emphasizes cyclical symptom patterns and diagnostic differentiation from other mental disorders [7, 8]. In addition, recruitment via social media may have resulted in a self-selected sample, potentially overrepresenting adolescents experiencing higher levels of distress or symptom burden. Thus, elevated PMDD prevalence in this sample likely reflects high levels of premenstrual symptom burden rather than PMDD diagnoses in a strict nosological sense. At the same time, recent epidemiological data indicate increasing rates of internalizing symptoms, such as depression and anxiety, particularly among adolescent girls [36]. It is therefore plausible that heightened premenstrual symptom reporting reflects an interaction between cyclical symptom patterns and broader mental health vulnerabilities during adolescence. To partially address this concern, the present study assessed positive mental health using the PMHS. Although this measure does not substitute for clinical diagnostic assessment, it provides an important contextual indicator suggesting that elevated premenstrual symptom burden cannot be explained solely by globally impaired mental health.

### Validity of the PMS-I-Y

Another aim of the study was to examine the factorial validity of the PMS-I-Y in adolescents. Exploratory analyses were first conducted to examine the underlying structure of the PMS-I-Y in this adolescent sample. The EFA results supported the presence of a dominant general factor, while solutions with two or more factors showed only limited differentiation between psychological and functional domains, with moderate factor correlations and several cross-loadings. A three-factor solution did not yield a meaningful additional dimension. These findings converge with the bifactor model and further support the interpretation that premenstrual symptom-related impairment in adolescents is primarily characterized by a general burden dimension with limited evidence for clearly separable subdomains. Exploratory item-reduction analyses further showed that removing items with weak loadings or cross-loadings modestly improved model fit, particularly in a 14-item solution. However, because this procedure was data-driven and could compromise content validity, these findings should be interpreted as preliminary. The original 18-item version was therefore retained, and potential short-form development should be examined in independent validation samples. These exploratory findings were subsequently supported by the confirmatory analyses. Both a two-factor model and a bifactor model were tested, with results indicating a strong general factor of premenstrual symptom burden alongside secondary functional and psychological dimensions. From a developmental and interpretive perspective, this finding suggests that adolescents may experience premenstrual symptoms as a more global burden affecting emotional well-being and daily functioning simultaneously, rather than as clearly separable psychological and functional domains. From a clinical perspective, this suggests that total scores assessing overall symptom-related burden may be more informative for screening purposes in adolescent populations than relying on narrowly defined subscales. Convergent and discriminant validity further provided preliminary support for the PMS-I-Y as a potentially useful screening tool for assessing premenstrual impairment in youth. Overall, the exploratory and confirmatory findings converge in supporting the interpretation that premenstrual symptom-related impairment in adolescents is primarily characterized by a general burden dimension with limited evidence for clearly separable subdomains.

### Implications

Consistent with previous research, adolescents in this study reported premenstrual distress patterns broadly comparable to those observed in adult samples [37, 38]. However, while earlier studies emphasized functional impairments such as social and academic disruptions [10, 39], participants in the present sample reported psychological impairments – such as difficulties managing stress, negative emotions, and intrusive thoughts – as more burdensome. This finding highlights the central role of emotional and cognitive symptoms in adolescent PMS and underscores the need to integrate mental health-oriented prevention and intervention strategies, such as emotion regulation training and resilience-building approaches, into adolescent-focused care models. Furthermore, the findings suggest that routine assessment of premenstrual symptom-related impairment may facilitate earlier identification of adolescents experiencing clinically relevant distress. The PMS-I-Y may therefore represent a useful screening instrument for research and potentially for clinical practice. Earlier identification of affected adolescents may support timely referral and intervention and contribute to reducing the long-term psychological and functional burden associated with premenstrual disorders. Nevertheless, further validation in independent and more representative adolescent samples is required before routine clinical implementation can be recommended.

### Strengths and limitation

The present study addresses an important gap by focusing on adolescents from German-speaking countries, a population underrepresented in PMS research. Strengths include the use of inclusive language, gender-diverse response options, and active youth involvement in the research design, enhancing relevance and ethical quality [40–42]. Several limitations should be acknowledged. First, reliance on retrospective self-report data may have inflated prevalence estimates relative to prospective daily symptom tracking, which is required for formal DSM-5 and ICD-11 diagnoses [8, 43]. In addition, recall bias, self-selection bias, and the broadened operationalization of PMS/PMDD thresholds likely contributed to elevated prevalence estimates and should be considered when interpreting the findings. Second, recruitment via social media may have introduced self-selection bias and limits the representativeness and generalizability of the findings. Third, differential diagnoses were not assessed, limiting conclusions regarding PMDD prevalence in a strict diagnostic sense. Fourth, we developed a classification approach to identify adolescents experiencing clinically relevant premenstrual symptoms and associated distress. Although this operationalization allowed us to differentiate between varying degrees of symptom burden, it may have broadened diagnostic thresholds and contributed to elevated prevalence estimates. Fifth, the adaptation process of the PMS-I-Y did not include formal pilot testing, cognitive interviewing, or content-validation procedures, which may limit conclusions regarding content validity. Sixth, although the mean age at menarche in this sample was approximately 12 years, the study included only adolescents aged 14 years and older, limiting conclusions about earlier developmental stages. Seventh, although the BFI-S provided a useful benchmark for examining discriminant validity with relatively distal personality traits, this approach may not fully capture more proximal aspects of psychological functioning. Furthermore, although exploratory and confirmatory analyses consistently supported a strong general factor, the differentiation between psychological and functional subdimensions was less clearly defined. Subscale interpretations should therefore be approached with caution. Finally, despite the use of an estimator appropriate for ordinal data, the limited number of response categories may still have affected parameter estimates.

### Recommendations

Future research should extend the present findings in several ways. First, including younger adolescents would help to better capture the early emergence and developmental trajectory of premenstrual symptom burden. Second, combining prospective symptom monitoring with qualitative approaches may provide a more comprehensive understanding of adolescents’ lived experiences and improve ecological validity. Third, future studies should include more proximal constructs – such as general mental health, somatic complaints, or quality of life – to further evaluate discriminant validity. Finally, explicitly addressing PMS and PMDD in non-binary and transmasculine populations remains a critical research priority, given evidence for elevated mental health vulnerabilities in these groups [44, 45].

## Conclusion

This study demonstrates that premenstrual symptoms are highly prevalent already in adolescence and often associated with substantial psychological burden, underscoring the need for greater attention to PMS and PMDD in this age group. The adolescent-adapted Premenstrual Impact Questionnaire (PMS-I-Y) shows promising preliminary psychometric properties for assessing overall premenstrual impairment in adolescents aged 14–18 years. The strong general impact factor suggests that the PMS-I-Y may be useful for preliminary screening and research, although further validation is needed before firm clinical recommendations can be made. Early, age-appropriate assessment and holistic management of premenstrual symptoms may help reduce stigma and support adolescent well-being, potentially preventing symptom persistence into adulthood.

## Data Availability

The datasets generated and/or analysed during the current study are not publicly available due to data protection regulations involving minors but are available from the corresponding author on reasonable request.
